# Altholactone Displays Promising Antimicrobial Activity

**DOI:** 10.3390/molecules16064560

**Published:** 2011-06-03

**Authors:** Fouad Al Momani, Ahmad S. Alkofahi, Nizar M. Mhaidat

**Affiliations:** 1 Faculty of Applied Sciences, Jordan University of Science & Technology, Irbid 22110, Jordan; 2 Faculty of Pharmacy, Jordan University of Science & Technology, Irbid 22110, Jordan

**Keywords:** altholactone, antimicrobial agents, plant natural product

## Abstract

The antimicrobial activity of altholactone, a naturally extracted styryllactone isolated from *Goniothalamus malayanus*, was determined against Gram positive (*S. aureus* ATTC 25923, *S. aureus* ATTC 25392, and *E. faecalis* ATTC 29212) and Gram negative (*E. coli* ATTC 35218, *S. typhi* ATTC 14023 and *P. aeruginosa* ATCC 27853) reference bacteria and against the fungus *C. albicans* ATTC 10231. Different concentrations of altholactone (0, 12, 25, and 50 μg/mL) were used. Results revealed that altholactone inhibited the growth of all tested microbes except *P. aeruginosa* ATCC 27853 in a dose-dependant manner, with the highest cytotoxic effects occuring at 50 μg/mL. The average of the inhibition zones of the different concentrations was between 0–30 mm. Furthermore, altholactone-induced antimicrobial activity against the more sensitive microbes was assessed by measuring the minimal inhibitory concentration (MIC). Results indicated that Gram positive (*S. aureus* ATTC 25923, *S. aureus* ATTC 25392, *and E. faecalis* ATTC 29212) cells were more sensitive to altholactone than Gram negative ones (*E. coli* ATTC 35218, *S. typhi* ATTC 14023). *C. albicans* showed moderate sensitivity. These results indicate that altholactone might be a potential antimicrobial agent, particularly in ciprofloxacin-refractory *S. aureus* and *E. faecalis* infections. Further investigations are required to illustrate the mechanism(s) by which altholactone produces its antimicrobial effects.

## 1. Introduction

**Figure 1:**
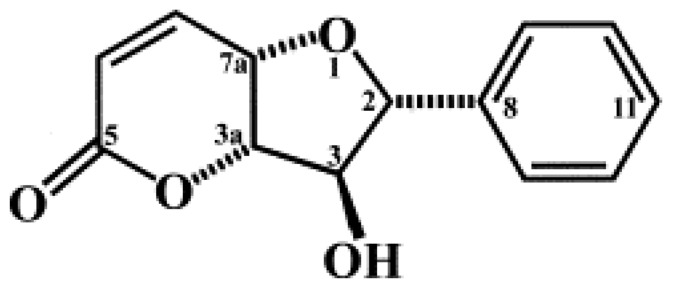
Chemical structure of altholactone.

The discovery, development and clinical use of antibiotics have substantially decreased public health hazards resulting from bacterial infections. However, there has been a parallel – and alarming – increase in bacterial resistance to existing chemotherapeutic agents [1]. Drug resistance with poor patient compliance, undesirable side effects, and the significant cost of combination therapy, reveals an intense demand for a therapeutic regimen having the same or higher beneficial properties of antibiotics, but with reduced side effects [2,3]. The purpose therefore of a new approach in treatment of infectious diseases comes from the identification of novel effective compounds with potent and useful activities against microbes. Altholactone (Figure 1) is a naturally occurring compound which is extracted, isolated from *Goniothalamus* spp. (Annoneceae) hooks [4,5,6]. Three main classes of compounds were found in *Goniothalamus* spp, including styryl lactones, annonaceous acetogenins and alkaloids. Phytochemistry and bioactivity studies have shown that all three styryl lactones possess cytotoxic activities against several models of human transformed cells [7,8]. Among them, altholactone is the most cytotoxic styryllactone [9] Previous studies have shown that altholactone exhibits antiplasmodial and antimycobacterial activities [10]. In the present study, we extended these explorations to evaluate the antimicrobial effect of altholactone against Gram positive and Gram negative bacteria such as *E. coli* ATTC 35218, *E. faecalis* ATTC 29212, *P. aeruginosa* ATTC 9027, *S. typhi* ATTC 14023, *S. aureus* ATTC 25923 and *S. aureus* ATTC 25392, and the fungus *C. albicans* ATTC 10231. 

## 2. Results and Discussion

**Figure 2:**
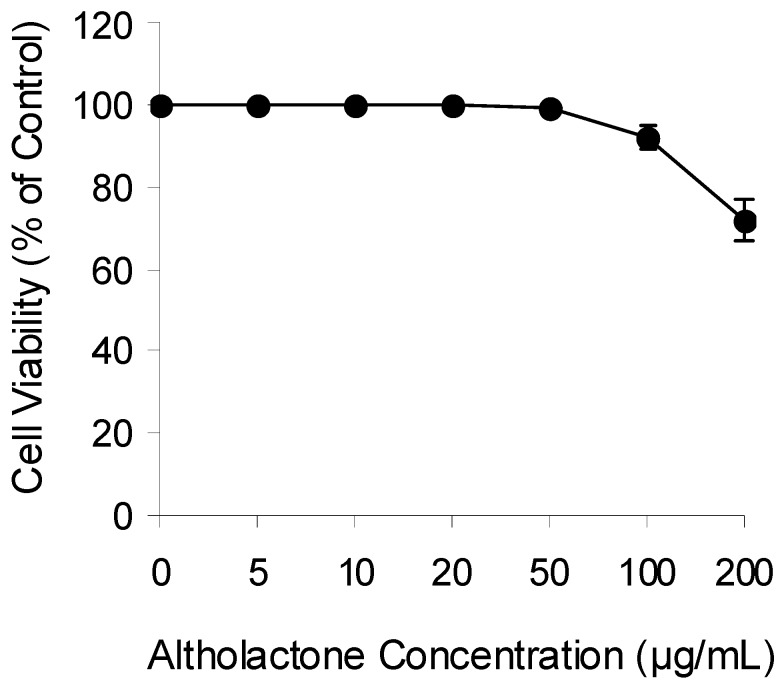
Study the cytotoxicity of altholactone in human normal fibroblasts. Cells were incubated with graded concentration of altholactone for 72 h and then analysed for cell growth using MTT assay. Each value represents the mean ± SE of three independent experiments performed with quadruplicate culture.

Plant natural products are used widely to induce cytotoxic effects against prokaryotic and eukaryotic cells. Previous studies have shown that altholactone displays cytotoxic effects against leukemia and breast cancer cell lines and against the aerobe *Mycobacterium tuberculosis* [10]. In view of the increased bacterial resistance, we extended these findings to evaluate the antibacterial activity of altholactone against different Gram positive, Gram negative and *C. albicans* reference strains. We first examined if altholactone induces cytotoxicity in normal human cells. FLOW2000 normal fibroblasts were used for this purpose. As shown in Figure 2, altholactone induces cytotoxicity against FLOW2000 cells in a dose-dependent manner. Altholactone did not become toxic when used at concentrations up to 50 μg/mL, therefore we used this concentration in our study. According to the results shown in Table 1, when *S. aureus* ATCC 25923 and *P. aeruginosa* ATCC 27853 were treated with a range of altholactone concentrations (0–50 µg/mL). Ciprofloxacin at 20 µg/disc and DMSO were used as positive and negative controls, respectively. Altholactone induced a dose-dependent antibacterial activity against *S. aureus* ATCC 25923 but not *P. aeruginosa* ATCC 27853. The optimal concentration of altholactone was 50 µg/mL at which the zones of inhibition ranged from 0 mm to 30 mm. An inhibition zone of 10 mm was chosen as representative of bacterial susceptibility to the compound. 

**Figure 3:**
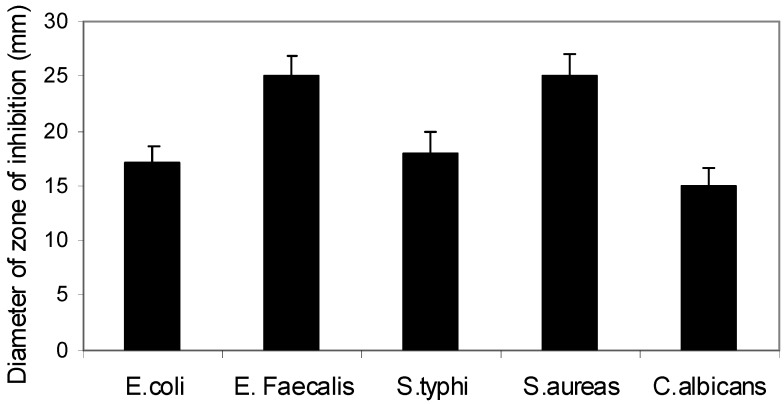
Altholactone inhibits growth of gram positive, gram negative, and fungi. Different bacteria were treated with altholactone at 50 μg/mL for 24 h before measurement of the diameter of inhibition zones. Data shown are the mean ± SE of three individual experiments.

Next, we extended this study sample to include other Gram positive (*S. aureas* ATTC 25392, and *E. faecalis* ATTC 29212) and Gram negative (*E. coli* ATTC 35218, *S. typhi* ATTC 14023) microbes and the fungus *C. albicans*. Results shown in Figure 3 reveal that altholactone at 50 μg/mL has good potency to inhibit the growth of the tested microbes. *P. aeruginosa* ATCC 27853 showed no sensitivity to altholactone compared to ciprofloxacin. Gram positive (*S. aureas* ATTC 25923, *S. aureas* ATTC 25392, and *E. faecalis* ATTC 29212) were more sensitive than Gram negative (*E. coli* ATTC 35218, *S. typhi* ATTC 14023) species to altholactone-induced toxicity. *C. albicans* showed a moderate degree of sensitivity. These differences in sensitivity among Gram positive (*E. faecalis* and *S. aureus*), Gram negative bacteria (*S. typhi* and *E. coli*) and *C. albicans* might be due to the nature of their respiration pathway or their oxygen requirements. The facultative anaerobic properties of *S. typhi* and *E. coli* makes them less sensitive to altholactone, an oxidative stress-inducing agent [12,13], or it could be due to the variation in their cell wall structure. 

Resistance of *P. aeruginosa* ATCC 27853 to altholactone or the overall less sensitivity of the Gram negative spp. could be due to the presence of some extrachromosomal DNA plasmids able to inactivate or degrade altholactone. Previous studies have shown that styryllactones, including altholactone, might selectively affect the mitochondrial membrane and/or mitochondrial respiratory system inducing oxidative stress [12,13]. In addition, altholactone has been shown to be DNA topoisomerases poison, which might result in the generation of toxic reactive oxygen species (ROS) [14]. Thus, varying degrees of sensitivity to altholactone might be due to different levels of ROS generated in different strain or due to different levels of oxidative stress mediators. These results were confirmed by measuring the MIC of altholactone against all bacterial strains. Different concentrations of altholactone were used (0–15 µg/mL). As shown in Table 2, different bacterial strains showed different sensitivity degrees to altholactone. 

The MIC values of Gram positive (*S. aureus* ATTC 25392, and *E. faecalis* ATTC 29212) species was lower than the MIC of Gram negative (*E. coli* ATTC 35218, *S. typhi* ATTC 14023) ones, indicating more sensitivity to altholactone. Taken together, these results indicated that altholactone might be a promising antimicrobial agent for use as an antiseptic compound against nosocomal microbes, as a fungicide for agricultural use or for the treatment of external dermatological infectious disorders, mostly against aerobic and facultative anaerobic microbes.

## 3. Experimental

### 3.1. Cell Viability Assays

The acute cytotoxic effect of altholactone on FLOW2000 cells was determined using MTT assays. Briefly, cells were seeded at 5000/well onto flat-bottomed 96-well culture plates and allowed to grow for 24 hours before the desired treatment. Cells were then labeled with MTT from the Vybrant MTT Cell Proliferation Assay Kit (Molecular Probes, Eugene, OR, USA) according to the manufacturer’s instruction and resulting formazan was solubilized with DMSO. Absorbance was read in a microplate reader (Bio-Rad, NY, USA) at 540 nm.

### 3.2. Well Diffusion Assay

Antimicrobial activity tests of altholactone and reference drugs was performed on Muller–Hinton Agar medium. Twenty milliliters of agar medium were poured into the plates to obtain uniform depth. The standard inoculums’ suspensions (10^6^ c.f.u./mL) were streaked over the surface of the media using sterile cotton swab to ensure the confluent growth of the organism. Altholactone was a generous gift from Professor A. Alkofahi (Jordan University of Science & Technology, Irbid, Jordan). Altholactone was dissolved in 5% DMSO. Five mm diameter wells were prepared in the medium and filled with 100 µL of different altholactone concentrations. Chloramphenicol at 10 µg/mL and ciprofloxacin at 20 µg/disc were used as positive controls. A nontoxic concentration of DMSO (0.1%) was used as a negative control. Next, inoculated plates were incubated at 37 °C for 24 h and the diameters of inhibition zones were measured. All the experiments were carried out in triplicate.

### 3.3. Determination of Minimum Inhibitory Concentration (MIC)

The minimum inhibitory concentration values were determined for the bacterial strains and *Candida albicans* which were sensitive to altholactone on Mueller–Hinton by the broth macro dilution method [11] The inoculation of the microbes was prepared from 4-hr-old broth cultures and suspensions were adjusted to standard turbidity (10^7^ c.f.u./mL). The compounds were dissolved in DMSO to obtain 20 mg/mL stock solutions. The stock solution was diluted with Muller–Hinton broth to give different concentrations (0, 12, 25 and, 50) µg/mL. Microbes were exposed to altholactone and Muller–Hinton broth to give a final concentrations of (10^7^ c.f.u./mL). Microbes treated with chloramphenicol (100 µg/mL), ciprofloxacin at 20µg/disc or 0.1% DMSO, were used as controls. The culture tubes were incubated at 37 °C for 24 h. The lowest concentration which did not show any growth of the tested organism on the culture medium after macroscopic evaluation was regarded as MIC.

## 4. Conclusions

The results indicate that altholactone might be a promising antimicrobial agent for use as an antiseptic compound against nosocomal microbes, as a fungicide for agricultural use, or for the treatment of external dermatological infectious disorders, mostly against aerobic and facultative anaerobic microbes. Further investigations are required to evaluate the toxicological profile of altholactone before reaching a definitive conclusion about altholactone’s use as an antimicrobial agent.

## Figures and Tables

**Table 1:** Altholactone induces a dose-dependent antimicrobial activity.

Chemical	Zone of inhibition (mm) ± SE	
Altholactone (µg/mL)	*S. aureus* ATCC25923	*P. aeruginosa* ATCC 27853
**0**	0	0
**12.5**	15 ± 1.5	0
**25**	24 ± 2.1	0
**50**	31 ± 2.5	1 ± 0
**Ciprofloxacin (20 µg/disc)**	0	20 ± 1.5
**Chloramphenicol (10 µg/mL)**	0	0

The data shown are the mean ± standard error (SE) of three individual experiments.

**Table 2:** Altholactone MIC against different microbes.

Microbe	MIC (µg/mL)
*S. aureus ATCC25392*	0.625
*S. aureus ATCC25923*	0.625
*E. faecalis*	0.625
*S. typhi*	1.25
*E. coli*	1.25
*P. aeroginosa*	>15
*C. albicans*	2.50

The data shown are the mean of three individual experiments.
